# Vickers Hardness and Microleakage of Coated Glass-Hybrid and High-Viscosity Glass Ionomer Systems in Thermocycled Conservative Class II Cavities: An In Vitro Study

**DOI:** 10.3390/dj14080499

**Published:** 2026-08-07

**Authors:** Chau Bao Ho, Truong Thien Tran, Lan Thi-Quynh Ngo

**Affiliations:** 1 Faculty of Dentistry, University of Medicine and Pharmacy at Ho Chi Minh City, Ho Chi Minh City 700000, Vietnam; chauhb@bvbinhthanh.org.vn (C.B.H.); tranthientruong@ump.edu.vn (T.T.T.); 2 Binh Thanh General Hospital, Ho Chi Minh City 700000, Vietnam

**Keywords:** class II restoration, glass-hybrid restorative system, high-viscosity glass ionomer cement, Vickers hardness, microleakage, thermocycling

## Abstract

**Background/Objectives**: Glass-hybrid and high-viscosity glass ionomer systems were developed to address limitations of conventional glass ionomer cements. This in vitro study compared post-thermocycling Vickers hardness and microleakage of a coated glass-hybrid system and a coated high-viscosity glass ionomer system in conservative Class II cavities. **Methods**: Thirty extracted human maxillary first premolars each received both systems in standardized mesio-occlusal and disto-occlusal cavities, enabling paired within-tooth comparisons. Following thermocycling and methylene blue immersion, Vickers hardness was measured on polished restoration cross-sections at occlusal and gingival locations. Microleakage was quantified using a continuous microleakage index and ordinal dye penetration scores. **Results**: The EQUIA Forte HT system had a higher tooth-level mean Vickers hardness than the Gold Label IX Extra system (95.05 ± 6.58 HV vs. 91.33 ± 7.50 HV), with a mean paired difference of 3.72 HV (95% CI, 0.32 to 7.11; *p* = 0.033; Cohen’s d_z_ = 0.41, 95% CI, 0.03 to 0.78). The location-specific mixed model also showed an effect of restorative system (*p* = 0.025), whereas neither testing location nor the system × location interaction was significant. No significant between-system difference was detected for the microleakage index or for either ordinal dye penetration score. **Conclusions**: Within the limitations of this in vitro study, the EQUIA Forte HT system had a modestly higher post-thermocycling tooth-level mean Vickers hardness than the Gold Label IX Extra system, although the clinical relevance of the difference remains uncertain. No between-system differences were detected in dye penetration outcomes.

## 1. Introduction

Posterior Class II restorations remain technically demanding because limited proximal access and the position of cervical margins complicate isolation, material adaptation, finishing, and marginal protection. The progressive reduction in dental amalgam use and growing demand for tooth-colored restorations have encouraged broader use of adhesive and ion-releasing materials in posterior cavities [1].

Glass ionomer cements (GICs) are water-based acid–base cements formed by reacting an ion-leachable fluoroaluminosilicate glass with an aqueous polyalkenoic acid [2]. They chemically interact with enamel and dentin, release and recharge fluoride, and permit relatively simple placement [2]. Conventional GICs remain limited for stress-bearing posterior restorations because of brittleness, low fracture toughness, limited wear resistance, and sensitivity to water gain or loss during early maturation [2,3]. Because maturation continues after the initial set, early surface protection may affect subsequent material behavior [3].

High-viscosity GICs and glass-hybrid restorative systems were developed to address some of these limitations. High-viscosity GICs typically use a higher powder-to-liquid ratio, which may enhance certain mechanical properties [3]. Glass-hybrid formulations in the EQUIA Forte family incorporate glass particles of varying sizes, including smaller reactive particles, along with high-molecular-weight polyacrylic acid [4,5]. In coated GIC-based systems, a resin-based surface coating may protect the immature cement surface by sealing surface irregularities and limiting early water imbalance and degradation [1,4].

In vitro studies have shown results that vary by material and protocol rather than a consistent mechanical advantage for glass-hybrid systems [1,5,6,7]. Some studies report that EQUIA Forte or related glass-hybrid materials have higher strength or surface hardness than comparators [5,6], whereas another found no fracture-toughness benefit for newer GICs in stress areas, though resin coating increased surface hardness [1]. More recent studies further indicate that microhardness is sensitive to coating, contamination, and thermal-aging conditions [4,8,9,10].

Clinical studies have reported acceptable performance of coated glass-hybrid or high-viscosity GIC restorations in selected Class II cavities, although esthetic surface changes, retention, and marginal adaptation remain concerns [11,12,13]. A recent systematic review and meta-analysis found very low-certainty evidence suggesting more favorable retention and marginal adaptation with bulk-fill resin-based restorations than with high-viscosity GIC-based restorations [14]. These findings warrant cautious, indication-specific interpretation; laboratory comparisons can characterize material behavior under controlled conditions but cannot establish clinical superiority.

Vickers hardness quantifies local resistance to indentation under a specified load and dwell time. It does not directly measure wear resistance, fracture toughness, marginal integrity, or clinical longevity [5,8]. Measurements at predefined occlusal and gingival locations permit regional comparison within a section but do not establish three-dimensional uniformity. Such comparison is relevant in Class II cavities because these regions differ in cavity geometry and finishing access.

Marginal sealing is also a concern in Class II restorations, particularly at proximal and cervical margins [15]. In dye penetration testing, restored teeth are exposed to a tracer before sectioning, and interfacial penetration is then assessed on the cut surfaces [16]. This method-sensitive surrogate does not reliably predict clinical outcomes, although it can provide a controlled comparative endpoint within a defined protocol [16]. Thermocycling subjects restorations to repeated temperature changes, although protocols vary widely and only partly reproduce oral conditions [17,18].

Evidence from paired within-tooth studies comparing coated glass-hybrid and high-viscosity GIC systems in conservative Class II cavities, while jointly assessing regional post-thermocycling hardness and marginal dye penetration, remains limited. The present study therefore compared the two systems using the same cavity geometry, conditioning procedure, and thermocycling protocol within the same teeth. Both base cements set through an acid–base reaction. Only their respective resin-based surface coatings were light-cured. The resulting inferences therefore concern the complete restorative systems rather than the separate effects of the base cement or coating. The null hypotheses were that the systems would not differ in tooth-level mean Vickers hardness, location-dependent hardness, microleakage index, or ordinal dye penetration scores.

## 2. Materials and Methods

### 2.1. Study Design

This in vitro study compared two coated glass ionomer-based restorative systems in standardized conservative Class II cavities prepared in extracted human maxillary first premolars. Each tooth received two cavities, one mesial-occlusal and one disto-occlusal, allowing both restorative systems to be tested within the same tooth in a paired design. The glass-hybrid restorative system comprised EQUIA Forte HT Fil used with EQUIA Forte Coat and is hereafter termed the EQUIA Forte HT system. The high-viscosity glass ionomer restorative system comprised GC Gold Label IX EXTRA CAPSULE used with GC G-Coat Plus and is hereafter termed the Gold Label IX Extra system. Both base restorative cements set through an acid–base reaction; light curing was applied only to their resin-based surface coatings. The two cement–coating combinations were evaluated as complete restorative systems, and the independent effects of the base cements and coatings were not assessed.

The primary outcome was tooth-level mean Vickers hardness after thermocycling. Hardness was assessed at a single post-treatment time point following thermocycling, dye immersion, sectioning, and surface finishing. No baseline hardness measurement or separate non-thermocycled control group was included. For each restorative system within each tooth, the tooth-level mean hardness was calculated as the arithmetic mean of the occlusal- and gingival-location hardness values; each location-specific value was the mean of three indentations. Secondary outcomes were location-dependent hardness, the exploratory absolute occlusal–gingival hardness difference, and microleakage at the tooth–restoration interface. The workflow included tooth selection, cavity preparation, paired restorative allocation, restoration and coating, 24 h storage in distilled water, thermocycling, dye penetration, sectioning, Vickers hardness testing, and microleakage assessment.

### 2.2. Ethical Considerations and Tooth Collection

The study protocol was approved by the Biomedical Research Ethics Committee of the University of Medicine and Pharmacy at Ho Chi Minh City, Vietnam (approval No. 1129/HĐĐĐ-ĐHYD on 13 November 2023). Extracted human maxillary first premolars were obtained from patients undergoing orthodontic extraction at the Faculty of Dentistry, University of Medicine and Pharmacy at Ho Chi Minh City. Only one tooth was obtained from each donor. The teeth were anonymized before laboratory use. The ethics committee waived the requirement for written informed consent because the study used anonymized discarded biological tissue and involved no patient interventions.

### 2.3. Tooth Selection, Cleaning, and Storage

The final experimental sample comprised 30 extracted human maxillary first premolars from donors aged 18–25 years. Teeth were eligible if the crown was intact and free of caries, previous restorations, and other visible loss of tooth structure. Teeth were excluded if they showed wear, previous endodontic treatment, cracks or fractures, fluorosis, enamel hypoplasia, dentin hypoplasia, or other conditions that could affect enamel or dentin mineralization. After extraction, the teeth were cleaned with hand scalers (Hu-Friedy Mfg. Co., LLC., Chicago, IL, USA) and then polished with fluoride-free pumice. The teeth were immersed in 5.25% sodium hypochlorite for 5 min, then stored individually in sealed containers filled with 0.9% saline at 4 °C until use. The storage solution was replaced every 3 days, and the teeth remained fully immersed throughout storage. The interval between extraction and cavity preparation did not exceed 1 month.

### 2.4. Sample Size

Because no directly comparable paired within-tooth Class II hardness data were available to support an expected effect size, the sensitivity of the planned two-sided paired *t*-test was evaluated using G*Power version 3.1.9.7 [19]. With 30 paired observations, α = 0.05, and 80% power, the minimum detectable standardized paired effect was d_z_ = 0.53, which is approximately medium under conventional benchmarks. This value represented a statistical detectability threshold rather than an expected effect size or a threshold of clinical importance. The design therefore comprised 30 paired within-tooth comparisons and 60 restorations.

### 2.5. Restorative Materials

All restorative, coating, and conditioning materials used in this study were manufactured by GC Corporation (Tokyo, Japan) and were used according to the manufacturer’s instructions. Details of the materials are presented in Table 1. Shade A3 was selected after visual comparison with the natural crown shade of the study teeth because it provided the closest available match among the shades available for both products. The same nominal shade was used for both restorative cements to standardize their appearance and reduce the possibility that material allocation could be inferred from shade. Shade was not investigated as an experimental factor.

### 2.6. Sample Allocation

Each tooth received two standardized conservative Class II cavities, one mesial-occlusal and one disto-occlusal. Allocation was performed using permuted block randomization with two possible configurations: In configuration A, the mesial cavity was assigned to the Gold Label IX Extra system (including GC Gold Label IX EXTRA CAPSULE used with GC G-Coat Plus) and the distal cavity to the EQUIA Forte HT system (including EQUIA Forte HT Fil used with EQUIA Forte Coat). In configuration B, the assignment was reversed. Blocks of size two were used, with one configuration A and one configuration B in each block. The order within each block was randomized in Microsoft Excel (Microsoft Corp., Redmond, WA, USA), and 15 blocks were generated to produce a balanced distribution of each material between mesial and distal cavities.

Allocation concealment was implemented using sequentially numbered, opaque, sealed envelopes prepared by an independent researcher who was not involved in cavity preparation, restoration, or outcome assessment. The allocation envelope was opened only after both mesial and distal cavities had been prepared and confirmed to meet the prespecified dimensional tolerances. Outcome assessors for Vickers hardness and microleakage were blinded to material allocation. Material-identifying marks placed on the teeth were concealed before outcome assessment. Blinding of the restorative operator was not feasible because the two restorative systems differed in handling and coating procedures. The order of restoration placement within each tooth was also prespecified in the allocation form and balanced between mesial and distal cavities to minimize cavity-order and handling-sequence effects.

### 2.7. Cavity Preparation

Each tooth was mounted in a gypsum block, with the cemento-enamel junction at least 4 mm above the gypsum base. Standardized conservative Class II cavities were prepared on the mesial and distal surfaces of each tooth using a high-speed handpiece with water cooling and a TF-21 tapered flat-end diamond bur (Mani, Tochigi, Japan). The bur had a maximum diameter of 1.6 mm and a tip diameter of 0.8 mm. A new bur was used after every three cavity preparations. Each preparation included an occlusal access portion and a proximal component. The cavity dimensions were standardized to 4.0 mm occlusogingivally, 4.0 mm buccopalatally, and 1.5 mm mesiodistally. The gingival wall was at least 1.0 mm coronal to the cemento-enamel junction. Dimensions were verified during preparation with a periodontal probe and a digital caliper, with a tolerance of ±0.2 mm. All cavities were prepared by one operator. Before the main experiment, the operator prepared pilot cavities in 10 teeth not included in the study. These pilot preparations were independently checked by a supervising examiner for conformity with the prespecified dimensional tolerances before the main experiment. After cavity preparation, all cavity dimensions were remeasured. A tooth was excluded before randomization if at least one dimension in either cavity fell outside the prespecified ±0.2 mm tolerance; only teeth with two conforming preparations proceeded to randomized allocation.

### 2.8. Restorative Procedures

A Tofflemire matrix retainer (YDM, Tokyo, Japan) with a 0.035 mm stainless-steel matrix band (TOR VM, Moscow, Russia) was used during restoration placement to restore the proximal contour of each Class II cavity. Both cavities in each tooth were conditioned using GC Dentin Conditioner for 20 s, rinsed with water, and gently air-dried without desiccating the cavity surface. The restorative capsules were activated and mixed for 10 s at approximately 4000 rpm using a Capsule Mixer CM-II (GC Corporation, Tokyo, Japan). The mixed material was inserted using a GC Capsule Applier (GC Corporation, Tokyo, Japan). Before insertion, the capsule was primed by pressing the applicator twice.

Extrusion of the restorative material into the cavity was initiated within 10 s after mixing. The working time from the start of mixing was 1 min 15 s for GC Gold Label IX EXTRA CAPSULE and 1 min 30 s for EQUIA Forte HT Fil at room temperature (approximately 25 °C). The restoration was shaped with a hand instrument. At 2 min 30 s after the start of mixing, the matrix was removed, and excess material was adjusted using a fine diamond finishing bur under water cooling.

Surface coating was then applied according to group allocation. GC G-Coat Plus was applied to GC Gold Label IX EXTRA CAPSULE restorations, and EQUIA Forte Coat was applied to EQUIA Forte HT Fil restorations. The coating was applied to the exposed restoration surface and restoration margins according to the manufacturer’s instructions. Each coating was applied with a microbrush and light-cured for 20 s using an LED light-curing unit (Led B, Woodpecker, Guilin, China; wavelength range, 420–480 nm). The irradiance was maintained at 850–1000 mW/cm^2^ and verified before each curing session using a Light Meter LM-1 radiometer (Woodpecker, Guilin, China). The distance from the light tip to the coated surface was maintained at approximately 1 mm during curing.

After restoration and coating, all specimens were stored in distilled water at room temperature for 24 h before thermocycling.

### 2.9. Thermocycling

Thermocycling was completed in six runs using the custom-built apparatus designed and assembled at the Faculty of Dentistry, University of Medicine and Pharmacy at Ho Chi Minh City, Ho Chi Minh City, Vietnam. Each run comprised five teeth (10 restorations), which were placed simultaneously in a single specimen basket and underwent the complete 5000-cycle protocol together. Each tooth contained one restoration from each restorative system; consequently, both systems were represented within every run.

Thermocycling was performed using a custom-built thermocycling apparatus designed and assembled at the Faculty of Dentistry, University of Medicine and Pharmacy at Ho Chi Minh City, Ho Chi Minh City, Vietnam. The apparatus was designed to automatically transfer a specimen basket between two water baths using a mechanical arm and timed relay system. All specimens were fully immersed in distilled water during thermocycling. The protocol consisted of 5000 cycles between 5 ± 1 °C and 55 ± 1 °C, with a dwell time of 25 s in each bath and a transfer time of 5 s. The hot bath was maintained at 55 °C using a thermostatically controlled water bath. The cold bath was maintained at 5 °C by adding ice as needed. Bath temperatures were monitored using a Testo 735-2 temperature-measuring instrument (Testo SE & Co. KGaA, Titisee-Neustadt, Germany) connected to two waterproof Type T immersion/penetration probes (model 0603 1293, Testo SE & Co. KGaA), with one probe immersed in each bath. The probes remained in place throughout each run, and temperature readings were recorded at 10-cycle intervals. All recorded readings remained within 4–6 °C for the cold bath and 54–56 °C for the hot bath. The dwell and transfer intervals were independently spot-checked with a stopwatch by a second researcher during each of the six runs and were consistent with 25 s and 5 s, respectively. The controller’s programmed cycle count and relay-timer settings were checked before each run. Operational characterization was limited to the temperature-monitoring and timing checks described above; no formal whole-system performance validation was performed, and no traceable calibration of the thermometer probe measurement chain was conducted as part of the study.

The 5000-cycle protocol was selected as a controlled laboratory thermal challenge based on prior microleakage research that used the same cycle count and temperature extremes [17]. Because no validated conversion between thermocycle count and clinical service time is available, no clinical aging duration was assigned to this protocol [18].

### 2.10. Dye Penetration and Specimen Sectioning

After thermocycling, the root apices were sealed with Peri Compound (GC Corporation, Tokyo, Japan). Two layers of nail varnish (Felina Deluxe, Francia Beauty Co., Ltd., Ho Chi Minh City, Vietnam) were applied to the entire tooth surface except for the restorations and a 1 mm zone of tooth structure around the restoration margins. The specimens were immersed in 2% methylene blue solution for 24 h. After dye immersion, excess dye was removed by rinsing under running water and gentle brushing for 10 min. The teeth were then air-dried.

The roots were sectioned 2 mm apical to the cemento-enamel junction, and the crowns were embedded in transparent self-curing epoxy resin prepared using EpoFix Resin and EpoFix Hardener (Struers, Ballerup, Denmark). Each crown was sectioned longitudinally in the mesiodistal direction through the center of both restorations using a slow-speed diamond saw (Struers, Ballerup, Denmark) under water cooling. The cutting disc was replaced after every two teeth. Sectioning produced one buccal and one palatal half, and each half contained cross-sections of both restorations. For each tooth, one half was randomly assigned to microleakage assessment and the other half to Vickers hardness testing. The cut surfaces were manually finished using 1000-grit abrasive paper (KOVAX Corporation, Tokyo, Japan). No sequential polishing with finer abrasives was performed. All sectioned halves were coded by an independent dentist before assessment.

### 2.11. Vickers Hardness Testing

Vickers hardness was measured on the 1000-grit-finished cross-sectional surfaces using a Future-Tech FV-300e hardness tester (Future-Tech Corp., Kawasaki, Japan) under a test force of 4.90 N (500 gf) and a dwell time of 20 s (HV 0.5/20). After sectioning and surface finishing, the specimens assigned to hardness testing were kept dry, and testing was initiated as soon as surface preparation was completed, without a planned intervening storage period. For each restoration, measurements were performed at two locations within the restored Class II cavity, one toward the occlusal aspect and one toward the gingival wall. The locations were positioned along the central occlusogingival axis and separated by approximately 2 mm.

Three indentations were placed at each location and averaged to obtain one location-specific hardness value. Indentations were separated by at least 2.5 times the indentation diagonal length and placed away from visible cracks, voids, restoration margins, and dye-stained defects. The three indentations were treated as technical replicates rather than independent observations. The indentation force, dwell time, and spacing were selected with reference to the general principles of ASTM E384-22 and adapted for sectioned dental restorative specimens [20].

Because methylene blue immersion preceded sectioning, all restorations underwent the dye-immersion step before the cross-sectional test surfaces were created. Hardness was then measured on newly sectioned, 1000-grit-finished surfaces. Both restorative systems followed the same sequence of thermocycling, dye immersion, sectioning, surface finishing, and hardness testing. The examiner was blinded to material allocation.

### 2.12. Microleakage Assessment

Microleakage was assessed on the sectioned half, which contained cross-sections of both restorations. The specimens were examined using a stereomicroscope (Olympus SZX10, Olympus, Tokyo, Japan) at ×16 magnification. Images were captured using a Lumenera Infinite 1-3C digital camera (Lumenera, Ottawa, Canada) and Infinite Capture software (Lumenera, Ottawa, Canada). Linear measurements were performed in ImageJ 1.38e (National Institutes of Health, Bethesda, MD, USA) after calibration with a 1 mm scale under the same imaging conditions.

Dye penetration was quantified along the tooth–restoration interface on the selected section. Axial-wall penetration was defined as dye progression from the occlusal cavosurface margin along the axial-wall tooth–restoration interface toward the axiogingival line angle. Gingival wall penetration was defined as dye progression from the gingival cavosurface margin along the gingival wall interface toward the same line angle. Stained length was measured separately within these two predefined, non-overlapping interface segments. The total evaluated interface length was defined as the sum of the full axial-wall and gingival wall interface-segment lengths extending from their respective cavosurface margins to the axiogingival line angle. For each measurement round, the microleakage index was calculated as the sum of the axial-wall and gingival wall stained lengths divided by the total evaluated interface length. The index ranged from 0 to 1, with higher values indicating a greater proportion of stained interface.

Ordinal dye penetration scores were recorded separately for axial-wall and gingival wall penetration using predefined criteria informed by ISO/TS 11405:2015 and adapted for the present conservative Class II cavity model (Table 2) [21]. Representative annotated stereomicroscopic images illustrating the measurement scheme and ordinal dye penetration scoring criteria are provided in Supplementary Figure S1.

Microleakage measurements and scoring were performed by an examiner blinded to material allocation. Continuous measurements were repeated after a three-week interval, and the examiner had no access to the first measurement set during the second round. For each round, the microleakage index was calculated independently. The mean of the two measurement rounds was used as the microleakage index for each restoration. Intra-rater reliability between the two rounds was assessed using intraclass correlation coefficients for continuous measurements.

Ordinal scores were also assigned twice with a three-week interval. Intra-rater reliability for ordinal scoring was assessed using quadratic-weighted kappa statistics. When the two scores differed, the original image was re-evaluated under blinded conditions, and a resolved final score was assigned before statistical analysis.

### 2.13. Statistical Analysis

Data management was performed using Microsoft Excel 365 (Microsoft Corp., Redmond, WA, USA), and statistical analyses were conducted in R version 4.4.2 (R Foundation for Statistical Computing, Vienna, Austria). The tooth was considered the experimental unit for all between-system comparisons. All tests were two-sided, with α = 0.05. The paired comparison of tooth-level mean Vickers hardness was designated as the primary analysis. All other outcome analyses were considered secondary or exploratory.

For each restoration, mean Vickers hardness was calculated as the average of the occlusal and gingival location means. Normality of the within-tooth differences was assessed using the Shapiro–Wilk test and Q–Q plots. Mean hardness was compared between restorative systems using a paired *t*-test and reported as mean ± standard deviation, mean paired difference, 95% confidence interval (CI), *t* statistic, and *p* value. Cohen’s d_z_ was calculated as the mean within-tooth difference divided by the standard deviation of the paired differences. The 95% confidence interval for d_z_ was obtained by inverting the noncentral t distribution.

Location-specific hardness, rather than individual indentation values, was analyzed using a linear mixed-effects model. Restorative system, testing location (occlusal or gingival), and their interaction were included as fixed effects, with random intercepts for tooth and for restoration nested within tooth. The model was fitted using the lme4 package. Fixed effects were evaluated using Type III Wald chi-square tests with sum-to-zero contrasts, as implemented in the car package. Estimated marginal means and 95% confidence intervals for each system-by-location combination were obtained using emmeans. Model assumptions were assessed by inspecting residual-versus-fitted and Q–Q plots.

The absolute occlusal–gingival hardness difference was calculated for each restoration and analyzed as an exploratory measure of regional hardness variation. Between-system values were compared using a paired *t*-test after assessment of the within-tooth differences as described above. Results were reported as mean ± standard deviation, mean paired difference, 95% confidence interval, and *p* value.

The microleakage index was summarized using medians, interquartile ranges, ranges, and mean ± standard deviation. Between-system differences were evaluated using an exact two-sided Wilcoxon signed-rank test. The Hodges–Lehmann estimate of the paired location shift was reported with a 95% confidence interval obtained from 10,000 paired bootstrap resamples at the tooth level. Final ordinal dye penetration scores were summarized as frequencies and percentages and compared using two-sided Wilcoxon signed-rank tests with asymptotic approximation, tie correction, and no continuity correction because ties and zero differences precluded exact calculation. Ordinal scores were not averaged.

Intra-rater reliability was assessed using single-measure two-way mixed-effects ICCs for absolute agreement for continuous microleakage measurements and quadratic-weighted kappa statistics for ordinal scores. Confidence intervals for reliability estimates were obtained from 10,000 percentile cluster-bootstrap resamples at the tooth level, with both restorations from each sampled tooth retained in every resample. No multiplicity adjustment was applied across secondary or exploratory analyses; accordingly, their *p* values were interpreted as supportive rather than confirmatory.

## 3. Results

Thirty-six otherwise eligible teeth underwent cavity preparation. Six were excluded before randomization because at least one dimension in either prepared cavity fell outside the prespecified ±0.2 mm tolerance. The remaining 30 teeth were randomized, and all 60 restorations were included in the analysis. Each tooth received both restorative systems, with balanced mesial/distal allocation (15 mesial and 15 distal restorations per system). No restorations failed, were excluded, or were replaced after allocation. No values were missing, and all microleakage indices and ordinal dye penetration scores were within their predefined valid ranges.

### 3.1. Vickers Hardness After Thermocycling

**Figure 1:**
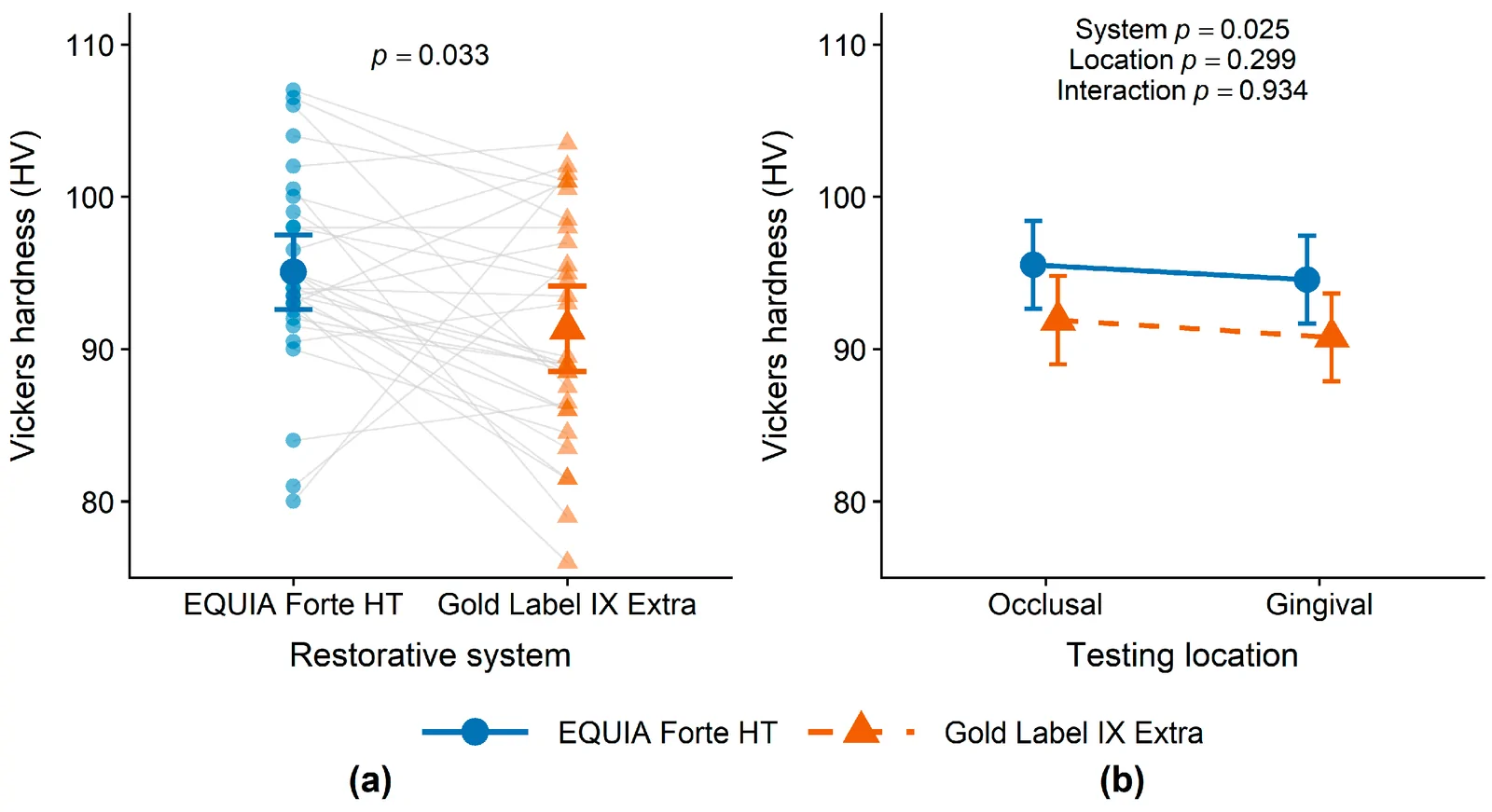
Vickers hardness after thermocycling. (**a**) Paired tooth-level mean Vickers hardness of the EQUIA Forte HT system and the Gold Label IX Extra system. Each light-gray line connects paired restorations from the same tooth. Colored symbols show individual observations, and larger symbols with error bars show the group means and 95% CI. (**b**) Location-dependent Vickers hardness at occlusal and gingival testing locations. Points and error bars represent estimated marginal means and 95% CI from the linear mixed-effects model. The *p* values in panel (**b**) were obtained from Type III Wald chi-square tests.

The mean Vickers hardness was 95.05 ± 6.58 HV for the EQUIA Forte HT system and 91.33 ± 7.50 HV for the Gold Label IX Extra system. The mean paired difference was 3.72 HV (95% CI, 0.32 to 7.11; t(29) = 2.24; *p* = 0.033), corresponding to Cohen’s d_z_ = 0.41 (95% CI, 0.03 to 0.78), with higher values for the EQUIA Forte HT system (Table 3; Figure 1a).

In the secondary location-dependent hardness analysis, the restorative-system effect met the nominal significance threshold (Wald χ^2^(1) = 5.02, *p* = 0.025), whereas testing location (Wald χ^2^(1) = 1.08, *p* = 0.299) and the restorative system × testing location interaction (Wald χ^2^(1) = 0.007, *p* = 0.934) did not (Figure 1b). Location-specific descriptive hardness values are shown in Table 3.

The absolute occlusal–gingival hardness difference was 7.23 ± 4.44 HV for the EQUIA Forte HT system and 5.80 ± 4.03 HV for the Gold Label IX Extra system. The mean paired difference was 1.43 HV (95% CI, −0.66 to 3.53; *p* = 0.173) (Table 3).

### 3.2. Microleakage and Dye Penetration After Thermocycling

**Figure 2:**
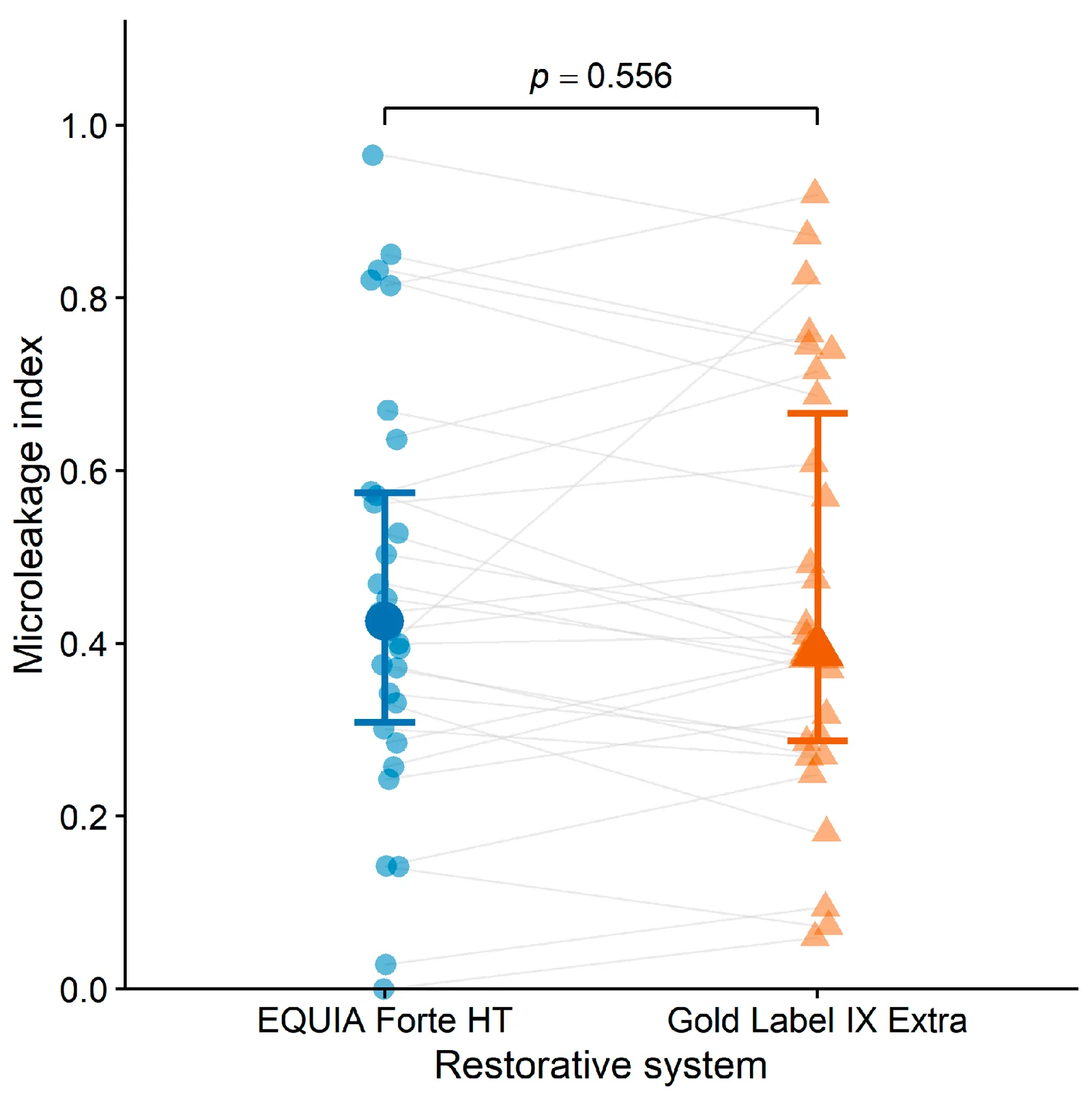
Microleakage index after thermocycling. Each light-gray line connects paired restorations from the same tooth. Colored symbols represent individual observations for the EQUIA Forte HT and Gold Label IX Extra systems. Large symbols with vertical bars indicate the median and interquartile range.

Descriptive statistics for the microleakage index are presented in Table 4A and Figure 2. The median microleakage index was 0.425 (IQR, 0.309–0.574) for the EQUIA Forte HT system and 0.391 (IQR, 0.287–0.667) for the Gold Label IX Extra system. The paired estimate is the Hodges–Lehmann estimate of the paired location shift (EQUIA Forte HT system − Gold Label IX Extra system). The exact Wilcoxon signed-rank test yielded *p* = 0.556. Zero microleakage was observed in one restoration with the EQUIA Forte HT system and in none of the Gold Label IX Extra restorations.

Resolved final ordinal scores were used for the analysis of axial-wall and gingival wall dye penetration. Their distributions are shown in Table 4B. No significant between-system difference was detected for the axial-wall score (*p* = 0.843) or the gingival wall score (*p* = 0.782).

Reliability estimates for repeated microleakage measurements and ordinal dye penetration scores are provided in Supplementary Table S1; ICC(A,1) values ranged from 0.868 to 0.997, and quadratic-weighted kappa values ranged from 0.814 to 0.829.

## 4. Discussion

The principal finding was a modest between-system difference in Vickers hardness under the present thermocycling and specimen-processing protocol, whereas no between-system difference was detected in regional hardness variation or dye penetration outcomes. The null hypotheses were therefore only partially rejected. Because the cement–coating combinations were evaluated as complete restorative systems, the findings do not identify the independent contribution of the base cement or coating.

The mean paired difference of 3.72 HV was approximately 4.1% of the Gold Label IX Extra mean and represented a modest standardized effect (Cohen’s d_z_ = 0.41; 95% CI, 0.03 to 0.78). The observed effect was smaller than the d_z_ = 0.53 associated with 80% power in the design sensitivity analysis, and its confidence interval indicates uncertainty in magnitude. In the absence of an established clinically anchored threshold for between-system differences in Vickers hardness of glass ionomer restorations, the present data cannot determine whether the 3.72-HV difference is clinically important. Vickers hardness reflects local indentation resistance and does not directly establish improved wear resistance, fracture resistance, marginal stability, or restoration longevity [1,11,12,13,14]. Absolute hardness values are also protocol-dependent because specimen geometry, surface preparation, storage, coating, aging, and indentation conditions vary across studies [4,8,9,10,20]. The result should therefore be interpreted as a modest laboratory difference rather than evidence of clinical superiority.

Differences in formulation and coating offer plausible but untested explanations for the hardness result. Acid–base maturation, material formulation, and surface protection can influence the measured hardness of glass ionomer-based materials [1,2,3,4,5,8]. However, the base cements and their respective coatings differed simultaneously. Because these components were not varied independently, the observed difference cannot be attributed specifically to either the base cement or the coating.

Neither a testing-location effect nor a restorative system × testing location interaction was detected. Thus, the data do not support a difference in the occlusal–gingival pattern between systems, but they also do not establish equivalent regional behavior or greater hardness uniformity for either system. No between-system difference was detected in the exploratory absolute occlusal–gingival hardness difference. This interpretation is limited to the two predefined locations along the central occlusogingival axis. Vickers values are sensitive to surface preparation, test force, dwell time, and the tested plane [20], while storage and maturation can further affect the properties of glass ionomer cements [2,3].

No between-system differences were detected in the microleakage index or in the axial- and gingival wall dye penetration scores. This result does not establish equivalent marginal sealing or the absence of leakage, because no equivalence margin was prespecified and measurable dye penetration occurred in both systems. The modestly higher hardness of the EQUIA Forte HT system did not coincide with a detectable between-system difference in dye penetration under the present protocol. However, hardness and dye penetration assess distinct aspects of restorative behavior.

Dye penetration is a method-sensitive, indirect surrogate and does not directly predict postoperative sensitivity, secondary caries, retention, or clinical failure [16,21]. Small dye molecules may traverse interfacial pathways differently from bacteria or oral fluids, and assessment of one section samples only part of a three-dimensional interface. Marginal and internal adaptation assessments and bacterial penetration models provide complementary evidence but are not interchangeable with dye penetration [15,22].

Clinical studies have reported acceptable outcomes for coated glass-hybrid and high-viscosity GIC restorations in selected Class II cavities, though retention, fracture, surface quality, anatomical form, and marginal adaptation remain relevant concerns [11,12,13,14]. These data provide clinical context but neither validate the present 3.72-HV difference nor establish the clinical superiority of either restorative system.

From a clinical decision-making perspective, the present findings do not support selecting the EQUIA Forte HT system over the Gold Label IX Extra system solely on the expectation that its modestly higher hardness will improve wear, marginal integrity, or longevity. At most, the hardness result indicates slightly greater resistance to local indentation in the tested cross-section after this laboratory protocol. In conservative Class II cavities resembling the present model, no between-system difference in dye penetration was detected after thermocycling; however, this does not establish clinical interchangeability. Material selection should therefore continue to rely on clinical evidence and case-specific factors, including cavity extent, occlusal demand, isolation, proximal adaptation, and proper use of the complete cement–coating system [11,12,13,14].

The 5000-cycle regimen served as a controlled thermal challenge, selected for comparability with previous microleakage research [17], rather than as a surrogate for a defined duration of clinical service; no validated conversion between cycle count and clinical time is available [18]. Thermocycling simulated repeated temperature changes and water exposure but not concurrent mechanical fatigue, pH fluctuations, biofilm, dentinal fluid or pulpal pressure, or functional wear. The findings should therefore be interpreted as responses to the present laboratory protocol rather than as evidence of long-term Class II performance.

Several design features support the internal validity of this comparison. The paired within-tooth design reduced inter-tooth variability because each tooth received both restorative systems, and only one tooth was obtained from each donor. Allocation to mesial or distal cavities and the order of restoration placement were balanced and prespecified; allocation was concealed; outcome assessors were blinded; and the main laboratory procedures were standardized. Technical replicates for hardness were averaged rather than analyzed as independent observations, and ordinal dye penetration scores were treated as ordinal data. These features address several reporting and bias domains recommended for preclinical dental materials research, including planning, allocation, specimen preparation, and outcome assessment [23].

Several limitations constrain interpretation. The extracted-premolar model did not reproduce proximal contact pressure, occlusal loading, pulpal pressure, dentinal fluid flow, or clinical variability in matrix adaptation and isolation. Thermal aging was not combined with mechanical loading, pH cycling, biofilm challenge, or functional wear. All cavity preparations and restorations were performed by one operator; this reduced within-study procedural variability but did not assess operator-dependent reproducibility. Hardness was measured only after thermocycling and specimen processing. Without a pre-thermocycling baseline or a separate non-thermocycled cohort, the study cannot determine whether thermocycling changed hardness or whether the between-system difference was already present before aging. The custom thermocycler underwent operational temperature and timing checks, but the apparatus was not formally validated as an integrated system. The thermometer probe measurement chain lacked traceable calibration, and temperature was not logged continuously. These factors limit external reproducibility and certainty regarding the exact thermal exposure, although both systems were exposed together within every run. Dye penetration was evaluated on a single selected section, so leakage outside that plane was not captured [16,21]. The hardness test plane was created only after dye immersion and therefore was not directly exposed as an open cross-section; nevertheless, methylene blue had contacted the external restoration surfaces and margins. Without a dye-free hardness cohort, an indirect material-specific influence cannot be quantified or excluded. Finally, manual 1000-grit finishing and dry post-sectioning handling make the absolute hardness values specific to this specimen-preparation protocol [20].

Parallel non-thermocycled and dye-free hardness cohorts are needed to separate the potential effects of thermal challenge and dye exposure, while factorial designs varying the base cement and coating independently could clarify their respective contributions. Future studies should combine thermal aging with mechanical loading, pH cycling, simulation of dentinal fluid or pulpal pressure, and biofilm-based challenges. Multi-section or three-dimensional leakage assessments, bacterial penetration models, and interfacial microscopy could provide a more comprehensive evaluation of interfacial sealing after aging than a single dye penetration section [15,22]. Longer maturation periods and repeated aging intervals may clarify whether hardness and leakage behavior change as glass ionomer-based materials continue to mature [2,3]. Clinical trials remain necessary to determine whether laboratory hardness and dye penetration findings translate into meaningful differences in restoration survival, marginal adaptation, or secondary caries.

## 5. Conclusions

Within the limitations of this in vitro study, the EQUIA Forte HT system had a modestly higher tooth-level mean Vickers hardness than the Gold Label IX Extra system under the present thermocycling and specimen-processing protocol (mean paired difference, 3.72 HV; Cohen’s d_z_ = 0.41), but the clinical relevance of this difference remains uncertain. No effect of testing location or restorative system × location interaction was detected, and no between-system difference was detected in the exploratory absolute occlusal–gingival hardness difference. No between-system difference was detected in the microleakage index or either ordinal dye penetration score; this should not be interpreted as evidence of equivalent marginal sealing. The higher hardness of the EQUIA Forte HT system was not accompanied by lower dye penetration, and the present findings do not establish clinical superiority or predict long-term Class II performance.

## Figures and Tables

**Table 1:** Restorative materials used in the study.

Material	Category	Shade	Lot Number
EQUIA Forte HT Fil ^†^	Glass-hybrid restorative	A3	2209221
EQUIA Forte Coat ^†^	Resin-based coating	N/A	2304121
GC Gold Label IX EXTRA CAPSULE ^‡^	High-viscosity GIC restorative	A3	2210121
GC G-Coat Plus ^‡^	Resin-based coating	N/A	2305021
GC Dentin Conditioner	Dentin conditioner	N/A	2210151

^†^ Components of the EQUIA Forte HT system. ^‡^ Components of the Gold Label IX Extra system. N/A, not applicable.

**Table 2:** Ordinal scoring criteria for dye penetration along the tooth–restoration interface.

Assessment	Score	Criterion
**Axial-** **wall** **penetration**	0	No dye penetration
	1	Dye penetration limited to enamel
	2	Dye penetration extending beyond the dentinoenamel junction but not reaching the level of the pulpal floor
	3	Dye penetration reaching the pulpal floor or extending deeper along the tooth–restoration interface
**Gingival wall** **penetration**	0	No dye penetration
	1	Dye penetration extending no more than one-half of the gingival wall length
	2	Dye penetration extending beyond one-half of the gingival wall length but not reaching the axiogingival line angle
	3	Dye penetration reaching the axiogingival line angle or extending onto the axial-wall interface

Scores were treated as ordinal variables and were not averaged.

**Table 3:** Descriptive and paired Vickers hardness outcomes after thermocycling.

Outcome	EQUIA Forte HT	Gold Label IX Extra	Paired Difference	95% CI	*p* Value
Occlusal hardness, HV	95.53 ± 8.83	91.90 ± 9.01	—	—	—
Gingival hardness, HV	94.57 ± 6.70	90.77 ± 7.50	—	—	—
Mean hardness, HV	95.05 ± 6.58	91.33 ± 7.50	3.72	0.32 to 7.11	0.033
Absolute occlusal– gingival difference, HV	7.23 ± 4.44	5.80 ± 4.03	1.43	−0.66 to 3.53	0.173

Values are presented as mean ± standard deviation unless otherwise stated. Paired differences were calculated as EQUIA Forte HT system − Gold Label IX Extra system. —, not applicable; HV, Vickers hardness; CI, confidence interval.

**Table 4:** Microleakage index and ordinal dye penetration scores after thermocycling.

**(A). Microleakage Index**							
**Outcome**	**EQUIA** **Forte HT**	**Gold Label** **IX Extra**	**Paired** **Estimate**		**95% CI**		***p* Value**
Median (IQR)	0.425 (0.309–0.574)	0.391 (0.287–0.667)	0.012		−0.024 to 0.060		0.556
Range	0.000–0.965	0.059–0.919	—		—		—
Mean ± SD	0.457 ± 0.243	0.454 ± 0.240	—		—		—
**(B). Ordinal Dye Penetration Scores**							
**Assessment**	**Score**	**EQUIA Forte HT,** ***n* (%)**		**Gold Label IX Extra,** ***n* (%)**		***p* Value**	
**Axial-wall** **penetration**	0	2 (6.7%)		2 (6.7%)		0.843	
	1	11 (36.7%)		11 (36.7%)			
	2	10 (33.3%)		11 (36.7%)			
	3	7 (23.3%)		6 (20.0%)			
**Gingival wall** **penetration**	0	2 (6.7%)		1 (3.3%)		0.782	
	1	11 (36.7%)		11 (36.7%)			
	2	7 (23.3%)		11 (36.7%)			
	3	10 (33.3%)		7 (23.3%)			

(A). The paired estimate is the Hodges–Lehmann estimate of the paired location shift (EQUIA Forte HT system − Gold Label IX Extra system). The 95% CI was obtained from 10,000 paired bootstrap resamples at the tooth level. The *p* value was calculated using an exact two-sided Wilcoxon signed-rank test. (B). Ordinal scores were analyzed as paired ordinal data and were not averaged. *p* values were calculated using two-sided Wilcoxon signed-rank tests with asymptotic approximation and no continuity correction because ties precluded exact calculation.

## Data Availability

The raw data supporting the conclusions of this article will be made available by the authors on request.

## Supplementary Materials

The following supporting information can be downloaded at: https://www.mdpi.com/article/10.3390/dj14080499/s1, Figure S1: Schematic illustration and representative stereomicroscopic images of microleakage measurement and ordinal dye penetration scoring. Table S1: Intra-rater reliability of repeated microleakage measurements and ordinal dye penetration scores.

## Abbreviations

The following abbreviations are used in this manuscript:

CI	Confidence interval
GICs	Glass ionomer cements
HV	Vickers hardness
ICC	Intraclass correlation coefficient
IQR	Interquartile range
SD	Standard deviation
