# Supplementary material for: Vickers Hardness and Microleakage of Coated Glass-Hybrid and High-Viscosity Glass Ionomer Systems in Thermocycled Conservative Class II Cavities: An In Vitro Study
Source: Dent J (Basel). 2026 Aug 7;14(8):499. doi: 10.3390/dj14080499 (PMC13510681; doi:10.3390/dj14080499)
Supplement: Supplementary file 1 [file dentistry-14-00499-s001.zip › dentistry-4392857-supplementary.pdf]

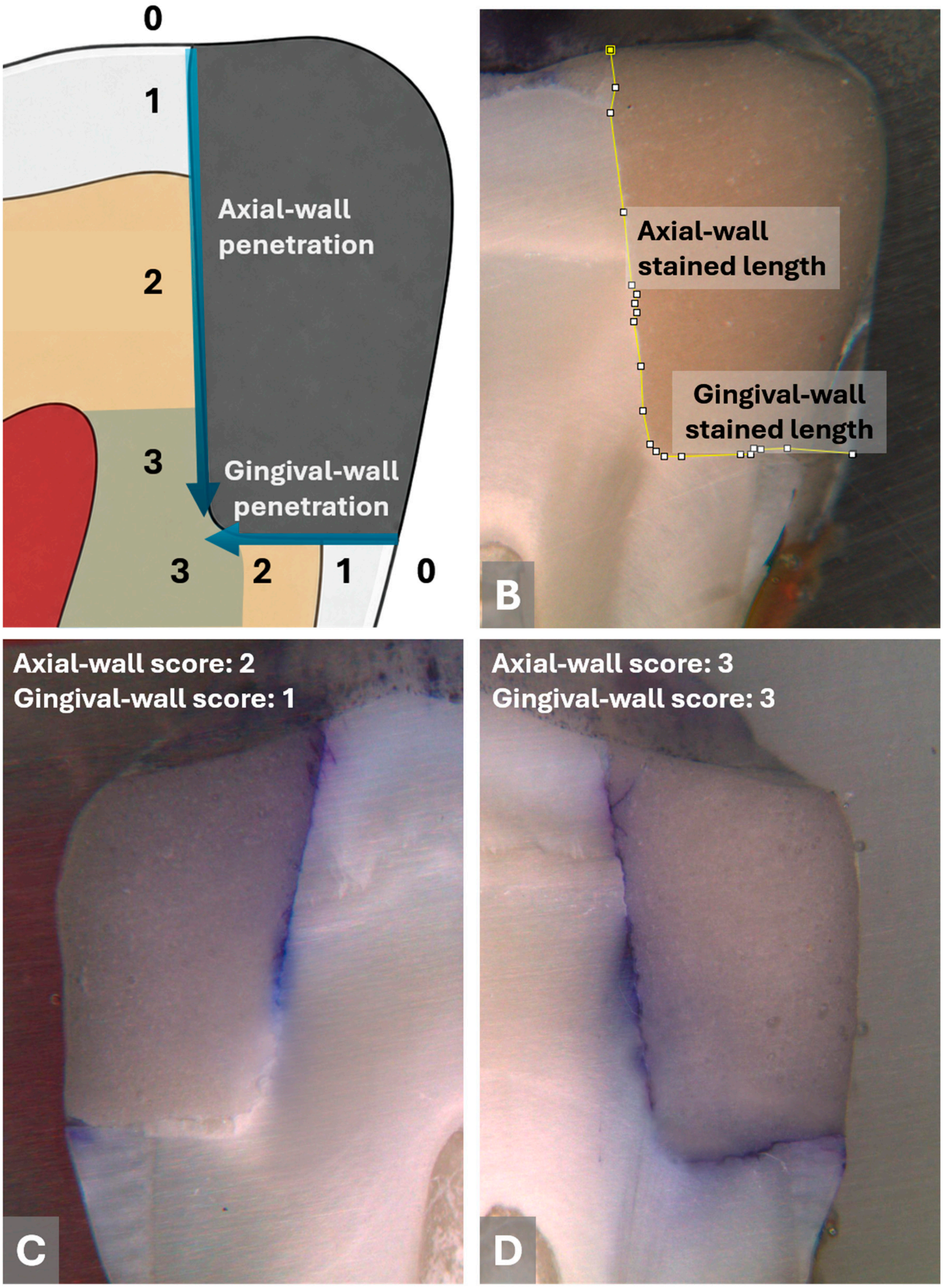

Figure S1. Schematic illustration and representative stereomicroscopic images of microleakage measurement and ordinal dye penetration scoring. (A) Schematic diagram of ordinal dye penetration scoring along the axial wall and gingival wall interfaces; a score of 0 indicates no dye penetration, whereas scores of 1–3 indicate increasing penetration depth along the tooth–restoration interface. (B) Representative annotated section showing the traced dye-stained lengths along the axial wall and gingival wall interfaces used to calculate the microleakage index. (C,D) Representative examples of ordinal dye penetration scores in sectioned restorations.

Table S1. Intra-rater reliability of repeated microleakage measurements and ordinal dye-penetration scores.

Measurement	Reliability statistic	Estimate	95% CI
Microleakage index	ICC(A,1)	0.997	0.996 to 0.998
Axial-wall stained length	ICC(A,1)	0.996	0.993 to 0.997
Gingival-wall stained length	ICC(A,1)	0.989	0.982 to 0.993
Total evaluated interface length	ICC(A,1)	0.868	0.803 to 0.908
Axial-wall dye-penetration score	Quadratic-weighted kappa	0.814	0.699 to 0.895
Gingival-wall dye-penetration score	Quadratic-weighted kappa	0.829	0.716 to 0.907

Reliability was assessed between the two microleakage measurement rounds. ICC(A,1) indicates a single-measure two-way mixed-effects intraclass correlation coefficient for absolute agreement. Quadratic-weighted kappa was used for ordinal dye-penetration scores. Confidence intervals were obtained using bootstrap estimation. CI, confidence interval.
